# Effectiveness of a primary care-based integrated mobile health intervention for stroke management in rural China (SINEMA): A cluster-randomized controlled trial

**DOI:** 10.1371/journal.pmed.1003582

**Published:** 2021-04-28

**Authors:** Lijing L. Yan, Enying Gong, Wanbing Gu, Elizabeth L. Turner, John A. Gallis, Yun Zhou, Zixiao Li, Kara E. McCormack, Li-Qun Xu, Janet P. Bettger, Shenglan Tang, Yilong Wang, Brian Oldenburg

**Affiliations:** 1 Global Health Research Center, Duke Kunshan University, Jiangsu, China; 2 Duke Global Health Institute, Duke University, Durham, North Carolina, United States of America; 3 The George Institute for Global Health, Beijing, China; 4 School of Health Sciences, Wuhan University, Wuhan, Hubei, China; 5 Peking University School of Global Health and Development, Beijing, China; 6 Melbourne School of Population and Global Health, The University of Melbourne, Victoria, Australia; 7 Vital Strategies, Jinan Representative Office, Beijing, China; 8 Department of Biostatistics & Bioinformatics, Duke University, Durham North Carolina, United States; 9 Beijing Tiantan Hospital, Capital Medical University, Beijing, China; 10 China Mobile Industry Institute, Chengdu, China; 11 Department of Orthopedic Surgery, Duke University, Durham North Carolina, United States of America; Columbia University, UNITED STATES

## Abstract

**Background:**

Managing noncommunicable diseases through primary healthcare has been identified as the key strategy to achieve universal health coverage but is challenging in most low- and middle-income countries. Stroke is the leading cause of death and disability in rural China. This study aims to determine whether a primary care-based integrated mobile health intervention (SINEMA intervention) could improve stroke management in rural China.

**Methods and findings:**

Based on extensive barrier analyses, contextual research, and feasibility studies, we conducted a community-based, two-arm cluster-randomized controlled trial with blinded outcome assessment in Hebei Province, rural Northern China including 1,299 stroke patients (mean age: 65.7 [SD:8.2], 42.6% females, 71.2% received education below primary school) recruited from 50 villages between June 23 and July 21, 2017. Villages were randomly assigned (1:1) to either the intervention or control arm (usual care). In the intervention arm, village doctors who were government-sponsored primary healthcare providers received training, conducted monthly follow-up visits supported by an Android-based mobile application, and received performance-based payments. Participants received monthly doctor visits and automatically dispatched daily voice messages. The primary outcome was the 12-month change in systolic blood pressure (BP). Secondary outcomes were predefined, including diastolic BP, health-related quality of life, physical activity level, self-reported medication adherence (antiplatelet, statin, and antihypertensive), and performance in “timed up and go” test. Analyses were conducted in the intention-to-treat framework at the individual level with clusters and stratified design accounted for by following the prepublished statistical analysis plan. All villages completed the 12-month follow-up, and 611 (intervention) and 615 (control) patients were successfully followed (3.4% lost to follow-up among survivors). The program was implemented with high fidelity, and the annual program delivery cost per capita was US$24.3. There was a significant reduction in systolic BP in the intervention as compared with the control group with an adjusted mean difference: −2.8 mm Hg (95% CI −4.8, −0.9; *p* = 0.005). The intervention was significantly associated with improvements in 6 out of 7 secondary outcomes in diastolic BP reduction (*p* < 0.001), health-related quality of life (*p* = 0.008), physical activity level (*p* < 0.001), adherence in statin (*p* = 0.003) and antihypertensive medicines (*p* = 0.039), and performance in “timed up and go” test (*p* = 0.022). We observed reductions in all exploratory outcomes, including stroke recurrence (4.4% versus 9.3%; risk ratio [RR] = 0.46, 95% CI 0.32, 0.66; risk difference [RD] = 4.9 percentage points [pp]), hospitalization (4.4% versus 9.3%; RR = 0.45, 95% CI 0.32, 0.62; RD = 4.9 pp), disability (20.9% versus 30.2%; RR = 0.65, 95% CI 0.53, 0.79; RD = 9.3 pp), and death (1.8% versus 3.1%; RR = 0.52, 95% CI 0.28, 0.96; RD = 1.3 pp). Limitations include the relatively short study duration of only 1 year and the generalizability of our findings beyond the study setting.

**Conclusions:**

In this study, a primary care-based mobile health intervention integrating provider-centered and patient-facing technology was effective in reducing BP and improving stroke secondary prevention in a resource-limited rural setting in China.

**Trial registration:**

ClinicalTrials.gov NCT03185858.

## Abstract 2

Lijing Yan and colleagues evaluate a mobile health intervention for secondary stroke prevention in China.

## Author summary

**Why was this study done?:**

- In low- and middle-income countries, there is an urgent need to develop effective and cost-effective approaches to strengthen primary healthcare system in noncommunicable disease management.
- Rural China suffers from an increasing burden due to stroke, with no existing effective strategy to manage community-dwelling stroke patients.
- Results from mobile health studies, usually not integrating health solutions for both primary healthcare providers and patients, were mixed with some demonstrating effectiveness while others had neutral findings.

**What did the researchers do and find?:**

- We conducted a cluster-randomized controlled trial in rural China to investigate whether a primary care-based integrated mobile health intervention could improve blood pressure (BP) control and secondary prevention of stroke.
- We identified individuals with stroke from 50 villages. Villages allocated in the intervention arm received both provider and patient-facing interventions over 12 months.
- There was a significant net reduction in systolic BP between the intervention and control groups, and the intervention also led to significant improvement in other health outcomes and reduction in hospitalization and mortality among stroke patients in rural China.
- The program was well implemented with relatively high fidelity and low cost (US$24.3 per participant per year).

**What do these findings mean?:**

- A primary care-based integrated mobile health intervention that aims to overcome both health system and individual barriers could lead to an improvement in BP control and secondary prevention of stroke in rural China.
- This model, with low program delivery cost, has the potential, when adapted to local contexts appropriately, to be relevant to other chronic conditions and in other resource-limited settings.

## Introduction

Since the Declaration of Alma-Ata in 1978, primary care strengthening has been identified as the key strategy for disease management and achieving universal health coverage [1]. Low-and middle-income countries (LMICs) bear the double burdens of long-standing infectious diseases and emerging noncommunicable chronic diseases (NCDs) [2]. However, primary care providers in LMICs often focus on infectious diseases and maternal and child health; thus, there is a lack of capacity to provide evidence-based essential primary care for patients with NCDs [3]. Several dozen trials have been conducted to evaluate various human-based or technological approaches to strengthen primary care for NCD control [4–9]. According to recent systematic reviews, these trials as a whole were effective in improving the quality of primary and community-based care, while results from mobile health (mHealth) technological interventions, mainly message-based programs, were inconclusive [8–11].

Our previous trials in rural China and India demonstrated that primary care-based multicomponent interventions to train, equip, and incentivize primary care providers were effective in changing providers’ behaviors and improving patient outcomes [12–14]. However, the interventions were not embedded in the existing healthcare system. In addition, in previous studies, the mHealth components targeted either providers [13] or patients [14] but were not integrated with each other. Many other trials suffered from similar problems [6] or did not utilize the rapidly evolving and promising mHealth technology at all [4,5].

To address these limitations, we designed an intervention program entitled “system-integrated and technology-enabled model of care (SINEMA)” [15]. This model strengthens the existing primary care workforce through training and support embedded in the entire healthcare system and integrates both provider-side and patient-facing mHealth technology. It is applicable to many types of NCDs, but we chose to focus on stroke management in rural China. Stroke is the leading cause of death and disability in rural China where primary care lacks the capacity to provide guideline-based essential care to stroke patients, and community-based management for secondary prevention of stroke is far from adequate [16,17]. We performed extensive feasibility and contextual research to adopt this model for stroke management in the setting of resource-limited areas in rural China [15,18,19]. We hypothesized that the SINEMA intervention would be more effective in improving blood pressure (BP) control and other health outcomes than usual care among stroke patients. To test this hypothesis, we conducted a cluster-randomized controlled trial in Northern China.

## Methods

### Trial design

The SINEMA study was an open-label, two-arm, cluster-randomized controlled trial with blinded assessment and analysis. We chose cluster-randomized design to implement the intervention at the cluster (village) level. Such a design could reduce contamination within clusters and enhance the feasibility of implementation. Over a 1-year period in preparation for the trial, we conducted extensive contextual field research on intervention design, technology development, and a 3-month pilot study in 4 villages [15,18,19]. We then conducted the main trial in 50 rural villages in rural China to evaluate the effectiveness of the intervention with embedded process evaluation and economic evaluation. Duration of the intervention was 12 months. The trial was registered on clinicaltrials.gov (NCT03185858). The study is reported according to the CONSORT guidance for reporting cluster-randomized trial (**S1 Checklist**) [20]. The trial protocol, technological development, and statistical analysis plan were published in detail [15,18,19,21]. We describe a condensed version below.

### Study setting and participants

The study was conducted in a rural region of Hebei Province, Northern China, where the incidence of stroke was 236.2 per 100,000 population, more than double the national average (109.7 per 100,000 population) [16,22]. In rural China, there is a 3-tier healthcare system including village clinics, township healthcare centers, and county hospitals [23,24]. Village clinics and township healthcare centers form the rural primary healthcare system and provide both general clinical care and the National Basic Public Health Services Program to residents in the region [23,25]. Village doctors who are not board-certified physicians but government-sponsored primary healthcare providers practice in village clinics. In general, they have high school or equivalent education with prescription rights for medicines in provincial essential medicine formularies. They are managed by and receive their supply from township healthcare centers. Although substantial investment and efforts had been made in primary healthcare strengthening since the Chinese healthcare reform in 2009, the resources remained substantially constrained, the quality of care is still suboptimal, and the utilization of primary care services has decreased [23–25].

In our study, eligible clusters were villages with a minimum population size of 1,500 and at least 1 village doctor who was willing to participate. The research team screened the eligible clusters among 218 villages from 8 townships in the region and selected 60 potentially eligible villages from 5 townships (as strata) where there were at least 10 clusters for further screening and recruitment. The main participant inclusion criteria were adults with a history of stroke diagnosed at the county- or higher-level hospitals and in a clinically stable condition with at least basic communication ability. Patients who were unable to get out of bed, had severe life-threatening diseases, or an expected life span shorter than 6 months were excluded. To ensure generalizability, mobile phone ownership or technology literacy was not a criterion for patient recruitment. Village doctors screened and invited potentially eligible patients in their villages to participate in the study. The research team conducted the final recruitment and consent process.

### Randomization and masking

Eligible villages were randomized in a 1:1 ratio to the intervention or the control arm with stratification by the township. A biostatistician who was not part of the study performed the randomization using a computer-generated random numbering system. Randomization allocation was not revealed to any staff during patient recruitment and assessment. Village doctors were informed only after patients’ baseline assessments were completed. Given the nature of the intervention, patients and health providers were not blinded to intervention assignment. Outcome assessors were kept unaware of the trial protocol and blinded throughout the study period to ensure the objectivity of assessment. Statisticians who were masked to intervention allocation conducted all statistical analyses, and allocation status was not revealed until all results were generated.

### Procedures

The SINEMA intervention package was developed with careful contextual research and pilot study lasting for 1-year long. The contents were consistent with China’s clinical guidelines for stroke prevention in a primary care setting [26] and tailored to the local context with special intervention focus on medication adherence and physical activities by considering the capacity and available resources [18,19]. In brief, the intervention included both provider-side components and patient-facing components and were supported by a digital health system consisted of an Android-based smart phone application—*SINEMA App*—for providers and linked with a voice messages system for patients (see **S1 Fig** for a diagram depicting the intervention design). The SINEMA *App* has been designed for multiple end-users including village doctors and township and county physicians and included multiple modules including patients’ profiles, follow-up visits, training, performance indicators, and follow-up visits reminders [19]. Aided by the SINEMA *App*, village doctors could collect, record, and retrieve patients’ information and follow-up history, and physicians from upper-tier hospitals could review the records and monitoring village doctors’ performance. The digital health system was linked with a third-party dispatching platform and a message bank containing more than 180 messages that we codesigned with clinical experts and local healthcare providers. These messages followed the same structure, was recorded in the local dialect, and were dispatched daily with different contents to participants. More detailed description on the development of the intervention package and digital health system could be found in previous publications [15,18,19].

After allocation of randomization, village doctors in the intervention arm were invited to a 1-day training session delivered by county hospital physicians who were trained by neurologists from a tertiary hospital. The training session covered the evidence-based use of essential medicines, skills for promoting patients’ behavior changes, and the use of *SINEMA App*. Each village doctor was also provided with a written intervention manual and an Android smartphone with the SINEMA App installed to support intervention delivery. Android instead of iOS (iPhone) was the smartphone operating system of choice due to its lower cost and widespread use among village doctors. A refresh training session was provided at the third month.

During the 12 months of the intervention period, village doctors delivered monthly follow-up visits to participants at the village clinics or participants’ homes, according to the standardized interventional plan. The follow-up visits covered BP monitoring, stroke symptom review, medication use assessment, and health education with a focus on medication adherence and physical activity. These 2 aspects were emphasized because our contextual research in the study region showed the importance of these behaviors among our study population and feasibility to intervene. Consistent with stroke management guidelines, [26] emphases on medication use were placed on 3 types of medicines (antiplatelet, statin, and antihypertensive). Village doctors were aided by the flow laid out in the *SINEMA App* to follow the standardized procedures. In each monthly follow-up visit, they gave participants a standardized single-sheet picture-rich handout listing their medications and exercise goals as a tool to illustrate the personalized recommendations. There was no cost for follow-up visits, but the cost of medications was borne primarily by the participants with partial coverage by social insurance, in consideration of the local norms and the need for sustainability and scalability.

Village doctors were provided with quarterly performance-based financial payment based on the quantity of services and bonuses for the top 5 village doctors determined by performance indicators that were generated from the *SINEMA App* and quality control measures provided by township physicians. Village doctors were encouraged to communicate with peers and township physicians through the App, phone calls, and the study’s virtual groups to share experience, seek clinical support, and provide feedback.

In addition to follow-up visits, participants who had access to their own or shared cell phones received 1 daily voice message at no cost to them over 12 months. Many patients were illiterate or not used to receive text messages even if they could read. Our pilot study in 4 villages found that voice calls were preferred over text messages [18]. Therefore, according to an algorithm designed based on our field research and pilot testing, [18] short voice messages—recorded in the local dialect—were automatically dispatched every morning with particular emphases on reminders and tips for medication adherence and physical activity.

In villages randomly assigned to the control arm, participants received usual care and village doctors continued their existing general clinical practices and the Basic Public Health Services. In the context of rural China, usual care involved patients seeking care in village clinics, township healthcare centers, or county hospitals, as needed. Some participants may also receive quarterly follow-up visits by village doctors if they had hypertension or diabetes, and receive general health education as such health promotion activities covered by the Basic Public Health Services were implemented widely across China [24].

### Outcomes

The primary outcome for patients was the 12-month change in systolic BP, analyzed as the difference between arms in the 12-month change in systolic BP from baseline to 12-month follow-up. The relatively short intervention duration and small sample size precluded the choice of stroke as the primary outcome. Systolic BP was chosen due to its well-established and significant impact on stroke recurrence and other cardiovascular events [16]. There were 7 prespecified secondary outcomes: diastolic BP, mobility functioning measured by the “timed up and go” test [27], physical activity based on the short-form International Physical Activity Questionnaire [28], health-related quality of life assessed by the EuroQol-5 Dimension-5L [29], and self-reported medication adherence to antiplatelet, statin, and antihypertensive measured separately by the 4-item Morisky Green Levine Scale [30]. Four prespecified exploratory outcomes included stroke recurrence and hospitalization, disability (modified Rankin Scale) [31], and mortality collected from questionnaires and medical and death records.

Outcome assessors were staff members from the Center for Disease Control and Prevention in a nearby county who were not involved in any of the program implementation. They were blinded on the intervention allocation and trained to follow a standard protocol to measure outcomes in exactly the same way in all villages and for all participants at baseline and 12 months. Data on patients’ self-reported information were collected through face-to-face interviews and recorded in an online survey platform (Qualtrics, Provo, Utah), with built-in skip patterns, logic checks, and quality control. BP was measured on the right upper arm with participants seated and after 5 minutes of rest, with an electronic BP monitor (Omron HEM-7052), validated by the European Society of Hypertension. Two measurements were taken. If the difference between the 2 systolic BP measures was larger than 10 mm Hg, a third measurement was conducted. The mean value of the only or the last 2 readings was used in all analyses.

### Sample size calculation and statistical analysis

We estimated that a sample of 1,250 prevalent stroke patients in 50 villages (with 25 villages per arm and on average 25 patients per village) would provide 83% power at a two-tailed 5% significance level to detect a 5-mm Hg mean difference in 1-year change of systolic BP between the intervention and control arms [32]. This estimation considered the cluster design and assumed a loss of 2 clusters per arm and loss of 1 patient per village, a standard deviation of change of 25 mm Hg, and an intracluster correlation coefficient of change of 0.04.

Analyses were conducted in the intention-to-treat framework, and all analyses were at an individual level with clusters and stratified design taken into account by following the prepublished statistical analysis plan [21]. The main analysis (minimally adjusted) compared the 1-year change in systolic BP between the intervention and control arms by using a mixed-effect model with a random intercept for the cluster (village) and a fixed effect for townships to account for the stratified design, baseline systolic BP, age, and sex. Restricted maximum likelihood was used together with the between-within method to calculate degrees of freedom [33]. Outliers were removed from change in systolic BP based on an a priori decision to remove those values which were more than 2 interquartile ranges above the third quartile or below the first quartile of the distribution [21]. Four types of sensitivity analyses were performed through changes to the minimally adjusted primary analysis model: (1) adjusting for baseline covariates that were identified as being imbalanced between the 2 arms or associated with lost-to-follow-up (fully adjusted analyses); (2) adjusting for covariates that were associated with baseline imbalance only; (3) adjusting for covariates that were associated with lost-to-follow-up only; and (4) with outliers included. We performed prespecified subgroup analyses (age, education, and duration since stroke event) by adding the subgroup variable and its interaction term with the intervention as fixed effects to the minimally adjusted model used in the main analysis.

Analysis of secondary and exploratory outcomes adopted a similar approach for the minimally adjusted models, fully adjusted models, and other sensitivity analyses. For continuous outcomes, the same mixed-effect modeling approach was used as described above. For binary outcome variables, the generalized estimating equation approach was used to obtain population-averaged intervention effects using a Poisson model with log-link and robust standard errors in order to obtain risk ratios [34,35]. An independent working correlation matrix was pre-specified because it was expected to provide greater stability when the cluster sizes were variable. In addition to all prespecified analyses in the statistical analysis plan [21], post hoc analysis on BP control rate (considering both systolic BP and diastolic BP) was also conducted to further estimate the clinical importance of the intervention. Risk differences for binary outcomes are computed directly from the outcome percentages at follow-up. All analyses were conducted in Stata version 15.1 (StataCorp, College Station, Texas) and were replicated by an independent statistician blinded to the results reported from Stata, using the *R* software (version 3.5.2) with the package of geeM [36] and Ime4 [37].

### Ethical statement

The study was approved by the Institutional Review Boards at Duke University, USA, Beijing Tiantan Hospital, and Duke Kunshan University, China. All participants (both providers and patients) provided written informed consent before participation. Cluster-level consent was provided by opinion leaders in the townships and villages.

## Results

### Recruitment and follow-up of study population

**Fig 1:**
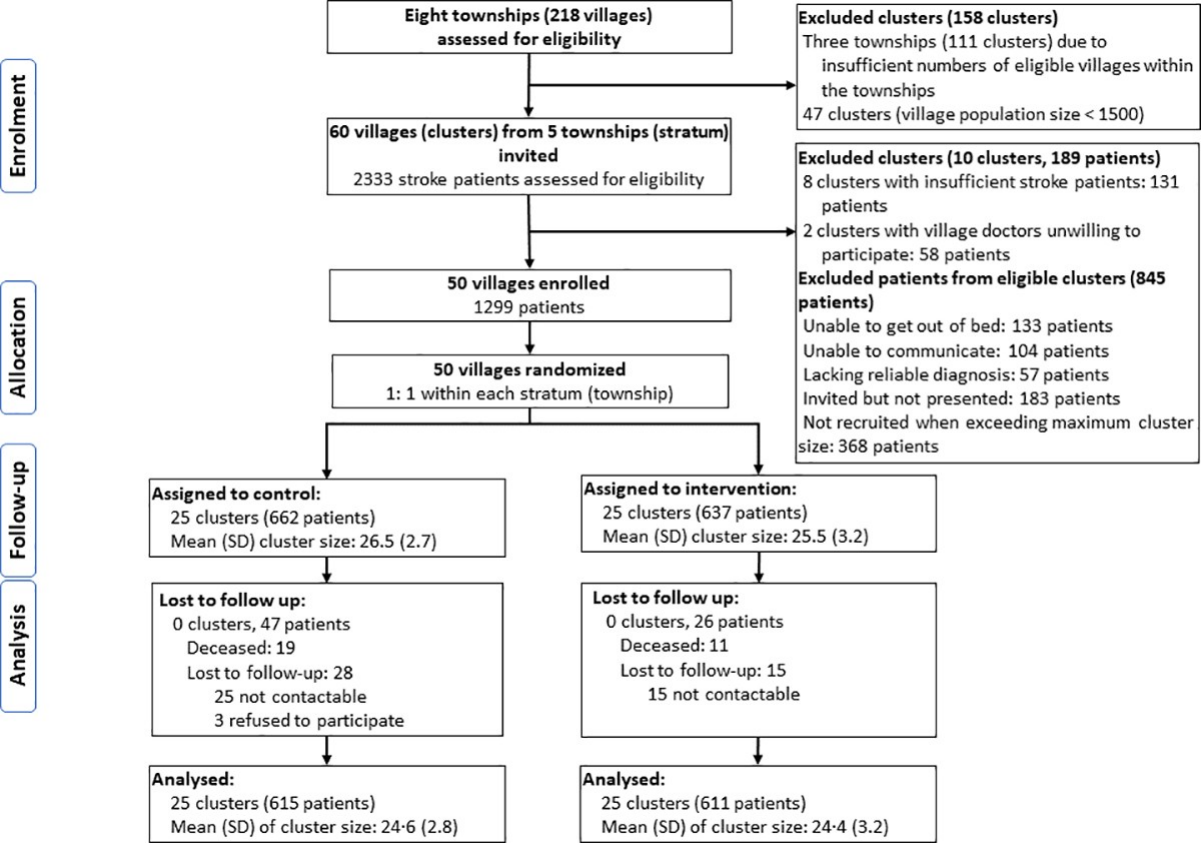
Flow diagram of trial participants.

Among 8 townships, we invited 5 townships where there were enough potentially eligible clusters to participate in the study and 60 village doctors performed eligibility screening among a total of 2,333 stroke patients from 60 villages (**Fig 1**). After excluding 10 ineligible villages and people who did not meet the inclusion criteria from the remaining villages, we recruited 50 villages with a total of 1,299 patients in the trial between June 23 and July 21, 2017. These 50 villages were randomized into the intervention arm (25 villages, 637 patients, and mean cluster size 25.5 patients per village [standard deviation–SD 3.2] and the control arm (25 villages, 662 patients, and mean cluster size 26.5 [SD 2.7]). After excluding those who died during the follow-up (*n =* 30, 2.3%) or lost to follow-up (*n* = 43, 3.3%), the final analyses included 1,226 participants.

### Baseline characteristics at patient and cluster level

Baseline characteristics at the patient level are summarized in **Table 1** and at the cluster and village doctor levels in **S1 Table**. For each of these 3 levels, the intervention and control arms were generally comparable. Patients in our trial were older adults (mean [SD] age 65.6 [8.2] years) who had low educational level (71.2% with no schooling or only a primary school education). The vast majority of participants (86.1%) had an ischemic stroke and had living with stroke for a median of 5.3 years (interquartile: 2.3 years, 9.8 years). Participants who died or were lost to follow-up (*n =* 73) were more likely (*p* < 0.05) to be male, younger, a current or former smoker, and in the control arm, and to have higher total family income and lower systolic BP at baseline (**S2 Table**).

**Table 1:** Baseline characteristics for the SINEMA trial at the patient level.

	Intervention (*n* = 637)	Control (*n* = 662)	Total (*n* = 1,299)
**Demographic characteristics and disease history**			
**Age, mean (SD), years**	66.2 (8.2)	65.2 (8.2)	65.7 (8.2)
**Sex, % female**	272 (42.7%)	281 (42.4%)	553 (42.6%)
**Education, n (%)**			
No schooling	264 (41.4%)	274 (41.4%)	538 (41.4%)
Some schooling or primary school only	182 (28.6%)	205 (31.0%)	387 (29.8%)
Above primary school	191 (30.0%)	183 (27.6%)	374 (28.8%)
**Marital status, n (%)**			
Married	526 (82.6%)	549 (82.9%)	1,075 (82.8%)
Widowed, divorced, or not married	111 (17.4%)	113 (17.1%)	224 (17.2%)
**Phone ownership, n (%)**			
No phone (may have a shared phone)	164 (25.7%)	159 (24.0%)	323 (24.9%)
Basic phone	435 (68.3%)	440 (66.5%)	875 (67.4%)
Smartphone	38 (6.0%)	63 (9.5%)	101 (7.8%)
Had none of the listed assets, n (%)*	28 (4.4%)	50 (7.6%)	78 (6.0%)
**Smoking status, n (%)**			
Current smoker	99 (15.5%)	122 (18.4%)	221 (17.0%)
Former smoker	130 (20.4%)	132 (19.9%)	262 (20.2%)
Never smoker	408 (64.1%)	408 (61.6%)	816 (62.8%)
**No (%) enrolled in NCD insurance benefits package**^†^	72 (11.3%)	96 (14.5%)	168 (12.9%)
**Stroke type, n (%)**			
Ischemic	555 (87.1%)	564 (85.2%)	1,119 (86.1%)
Hemorrhage	80 (12.6%)	96 (14.5%)	176 (13.6%)
Not specified	2 (0.3%)	2 (0.3%)	4 (0.3%)
**Stroke duration, years (median, interquartile)**			
Since the first event	5.3 (2.4, 9.8)	5.2 (2.3, 9.8)	5.3 (2.3, 9.8)
Since the latest event	3.2 (1.2, 6.8)	3.3 (1.1, 6.8)	3.3 (1.1, 6.8)
**Self-report diseases, n (%)**			
Hypertension	461 (72.4%)	436 (65.9%)	897 (69.1%)
Dyslipidemia	248 (38.9%)	271 (40.9%)	519 (40.0%)
Diabetes	113 (17.7%)	103 (15.6%)	216 (16.6%)
Heart Diseases	70 (11.0%)	54 (8.2%)	124 (9.5%)
**Outcomes at baseline**			
**Systolic blood pressure, mean (SD), mm Hg**	146.0 (20.9)	145.7 (23.7)	145.9 (22.4)
**Diastolic blood pressure, mean (SD), mm Hg**	78.0 (11.6)	79.7 (11.7)	78.9 (11.7)
**Health-related quality of life in utility, mean (SD)**^‡^	0.80 (0.2)	0.8 (0.21)	0.8 (0.2)
**Timed up and go, n (%) with completion time ≥14 s**^§^	324 (51.6%)	347 (53.1%)	671 (52.4%)
**Physical activity, median (Q1, Q3), MET minutes/week**	1,128.8 (346.5, 2,325.0)	924.0 (240.0, 2,304.0)	974.0 (297.0, 2,310.0)
**Medication use, n (%)**			
Antiplatelet	432 (67.8%)	420 (63.4%)	852 (65.6%)
Statin	158 (24.8%)	182 (27.5%)	340 (26.2%)
Antihypertensive medicines	522 (81.9%)	508 (76.7%)	1,030 (79.3%)
**Adherence to medications, n (%)**^††^			
Antiplatelet	275 (63.7%)	262 (62.4%)	537 (63.0%)
Statin	106 (67.1%)	110 (60.4%)	216 (63.5%)
Antihypertensive medicines	329 (63.0%)	316 (62.2%)	645 (62.6%)
**Moderate to severe disability, n (%)**^‡‡^	179 (28.1%)	173 (26.1%)	352 (27.1%)
**Stroke hospitalization in the past year, n (%)**	124 (19.5%)	132 (19.9%)	256 (19.7%)

MET, metabolic equivalents; NCD, noncommunicable chronic disease; SD, standard deviation.

*TV, refrigerator, air conditioner, and computer were listed as home assets in the questionnaire.

^†^NCD insurance package is only available for people enrolled in the health insurance system and with severe chronic diseases, through which people could get reimbursement of outpatient services at county hospital.

^‡^Health-related quality of life was measured by using EQ5D-5L and was converted into a utility score based on the Chinese value set. The utility score ranged from −0.4 to 1.

^§^Up and go test results were recorded in seconds during measurement and dichotomized into binary as ≥14 (indicating lower limb mobility) versus <14 s (higher limb mobility) based on previous literature.

^††^Medication adherence was only measured among participants who were taking the specific medicine based on 4-item Morisky Green Levine Scale.

^‡‡^Disability was measured by the modified Rankin Scale, and people who received a score above 3 were grouped into the “moderate to severe disability” group.

### Implementation fidelity and program delivery cost

The intervention was implemented with relatively high fidelity to the study protocol (**S3 Table**). All village doctors participated in the training adopted the *SINEMA App* for follow-up visits and utilized the support mechanisms. About 90.5% participants received 12 follow-up visits as planned. The daily voice messages were dispatched to 80.4% of participants in the intervention arm with access to cell phones, and on any given day, half (49.7%) of dispatched voice messages were successfully answered. The estimated annual cost of program delivery was about US$24.3 per patient (**S4 Table**).

### Effect of interventions on primary, secondary, and exploratory outcomes

**Fig 2:**
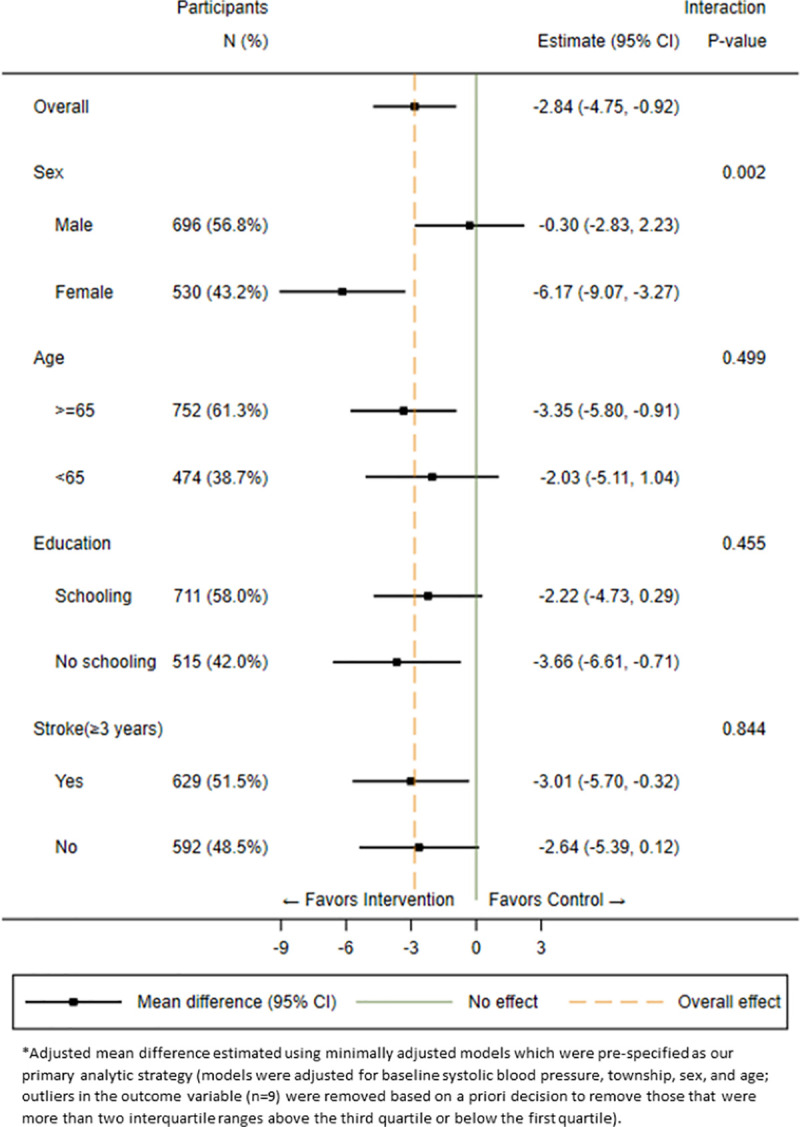
The adjusted mean difference in change in systolic blood pressure for the total population and by prespecified subgroups.

We observed a modest but significant intervention effect on systolic BP with a greater reduction in mean systolic BP among patients in the intervention arm (−7.1 mm Hg) compared with the control arm (−4.3 mm Hg) (adjusted mean difference: −2.8 mm Hg, 95% confidence interval [CI]: −4.8, −0.9, *p* = 0.005) within the primary minimally adjusted analytic model. The estimated intracluster correlation for this model was 0.001. The fully adjusted model for the primary outcome of change in systolic BP yielded a slightly higher adjusted mean reduction (−3.3 mm Hg, 95% CI: −5.2, −1.4) (**Table 2**). Results were robust and consistent within each level of the prespecific binary subgroup variables with exceptions of males (−0.3 mm Hg, 95% CI: −2.8, 2.2) and those less than 65 years old (−2.0 mm Hg, 95% CI: −5.1, 1.0) for which the between-arm differences were small (**Fig 2**). The additional analysis on BP control revealed that the intervention resulted in a 19% (95% CI: 8%, 30%) relative increase in the proportion of patients reaching the target of BP control (systolic BP < 140 mm Hg and diastolic BP < 90 mm Hg) (**S5 Table**).

**Table 2:** Minimally adjusted and fully adjusted results on primary, secondary, and exploratory outcomes.

	Intervention Arms		Minimally Adjusted Model*		Fully Adjusted Model**	
Outcomes	Intervention (*n* = 611)	Control (*n =* 615)	Estimate (95% CI) †	*p*-value	Estimate (95% CI)†	*p*-value
**Primary Outcome**						
Change in systolic blood pressure, mean (SD), mm Hg	−7.1 (18.5)	−4.3 (18.9)	−2.8 (−4.8, −0.9)^‡^	0.005	−3.3 (−5.2, −1.4)	0.001
**Secondary Outcomes**						
Change in diastolic blood pressure, mean (SD), mm Hg	−3.9 (9.6)	−2.3 (9.6)	−2.2 (−3.2, −1.3)	<0.001	−2.34 (−3.3, −1.4)	<0.001
Change in health-related quality of life score, mean (SD)^§^	0.01 (0.15)	−0.03 (0.14)	0.04 (0.01, 0.06)	0.006	0.04 (0.01, 0.06)	0.008
Change in physical activity, mean (SD), MET min/wk	1,203.9 (2,243.7)	750.9 (2,097.2)	528.2 (286.3, 770.1)	<0.001	490.2 (244.1, 736.3)	<0.001
Timed up and go (time of completion ≥14 s)^¶^	256 (43.7%)	298 (50.9%)	0.87 (0.77, 0.98)	0.023	0.87 (0.77, 0.98)	0.022
Medication adherence in Antiplatelets, n (%)^††^	308 (69.7%)	244 (66.7%)	1.03 (0.93, 1.14)	0.614	1.02 (0.92, 1.14)	0.658
Medication adherence Statins, n (%)^††^	133 (77.3%)	112 (62.9%)	1.21 (1.06, 1.38)	0.005	1.23 (1.07, 1.40)	0.003
Medication adherence Antihypertensives, n (%)^††^	383 (73.7%)	315 (66.5%)	1.10 (1.00, 1.22)	0.051	1.11 (1.00, 1.22)	0.039
**Exploratory Outcomes**						
Stroke Recurrence, n (%)	27 (4.4%)	57 (9.3%)	0.46 (0.32, 0.66)	<0.001	0.45 (0.31, 0.66)	<0.001
Stroke hospitalization in the past year, n (%)	27 (4.4%)	57 (9.3%)	0.45 (0.32, 0.64)	<0.001	0.44 (0.31, 0.64)	<0.001
Moderate to severe disability, n (%)^‡‡^	128 (20.9%)	186 (30.2%)	0.65 (0.53, 0.79)	<0.001	0.67 (0.55, 0.81)	<0.001
Death, n (%)^§§^	11 (1.8%)	19 (3.1%)	0.52 (0.28, 0.96)	0.036	NA^§§^	NA

CI, confidence interval; MET, metabolic equivalents; NA, not applicable; SD, standard deviation.

*Prespecified main analysis (minimally adjusted model): Adjusted for baseline outcome, township, sex, and age; removing outliers in the outcome variable (based on a priori decision to remove those that are more than 2 interquartile range above the third quartile or below the first quartile).

**Sensitivity analysis (fully adjusted model): Adjusted for baseline outcome, township, sex, age, variables noted to be differential by treatment arm at baseline (baseline diastolic blood pressure, having hypertension, having none of the assets asked about, taking antihypertensive medications), and loss to follow-up (baseline systolic blood pressure, annual household income, type of phone owned and smoking status); removing outliers in the outcome variable (based on a priori decision to remove those that are more than 2 interquartile ranges above the third quartile or below the first quartile).

^†^For continuous outcomes (systolic blood pressure, diastolic blood pressure, EQ5D-5L, physical activity), “estimate” refers to the differences between the arms in mean 1-year change in the outcome (control arm is the reference); for binary outcomes (timed up and go, medication adherence, stroke recurrence, stroke hospitalization, disability, and death), “estimate” refers to the risk ratio (control arm is the reference).

^‡^The intercluster coefficient is less than 0.001 for the model.

^§^Health-related quality of life was measured by using EQ5D-5L and was converted into a utility score based on the Chinese value set.

^¶^“Timed up and go test” results were recorded in seconds during measurement and dichotomized into binary as ≥14 (indicating lower limb mobility) versus <14 s (higher limb mobility) based on previous literature.

^††^Medication adherence refers to a perfect adherence with score of 0 based on the 4-item Morisky Green Levine Scale. Medication adherence was only measured among participants who were taking medicines. Medication adherence outcomes were not adjusted for baseline outcome, since the set of participants taking a given medication at baseline was not the same set taking the medicines at follow-up.

^‡‡^Disability was measured by the modified Rankin Scale, and people who received a score above 3 were grouped into the "moderate to severe disability" group.

^§§^The statistical model with death as the outcome was not adjusted for variables differential by the loss to follow-up, since those who died during the study were a subset of the group lost to follow-up.

We observed significant and meaningful beneficial effects of the intervention on 6 out of 7 prespecified secondary outcomes with a significantly greater reduction in diastolic BP, improvement in health-related quality of life, performance in “timed up and go” test, physical activity level, and medication adherence to statin and antihypertensives in the intervention arm (**Table 2**). The intervention arm also brought significantly fewer number of events in stroke recurrence (4.4% versus 9.3%; risk ratio [RR] = 0.46, 95% CI 0.32, 0.66; risk difference [RD] = 4.9 percentage points [pp]), hospitalization (4.4% versus 9.3%; RR = 0.45, 95% CI 0.32, 0.62; RD = 4.9 pp), disability (20.9% versus 30.2%; RR = 0.65, 95% CI 0.53, 0.79; RD = 9.3 pp), and death (1.8% versus 3.1%; RR = 0.52, 95% CI 0.28, 0.96; RD = 1.3 pp. Results were consistent in 3 additional sets of sensitivity analyses (**S6 Table**).

## Discussion

### Principal findings and interpretations

In this cluster-randomised controlled trial conducted among stroke patients in rural China, BP control was significantly improved through the primary care-based integrated mHealth intervention. The intervention also improved 6 out of 7 prespecified secondary outcomes and all exploratory outcomes on stroke recurrence, hospitalization, disability, and mortality, at an annual cost of less than US$24 per patient.

The intervention group experienced a 7.1-mm Hg reduction in the primary outcome of systolic BP, and the control group also had a 4.3-mm Hg reduction; thus, the magnitude of the net between-group difference was modest (−2.8 mm Hg). Plausible explanations for this finding included (1) a deliberate interventional design that relied on existing resources, e.g., not providing free medicines like previous trials [4]; (2) the lower BP level of 145.9 mm Hg and higher treatment rate (79.3%) at baseline compared to other trials [4,6]; and (3) improved implementation of the government-funded nationwide basic public health services [23,24] leading to the considerable reduction in the control group. Although the observed difference of 2.8 mm Hg was smaller than the 5-mm Hg estimate in the a priori power calculation, it was statistically significant (*p* = 0.005), possibly due to the smaller than hypothesized intracluster correlation coefficient or smaller than hypothesized standard deviation. More importantly, even a net reduction of only 2 mm Hg in systolic BP is clinically meaningful as ample research has shown that it can lead to a 7% to 10% decrease in fatal and nonfatal cardiovascular risks [38–40]. Additionally, our study was not a BP trial but a multicomponent stroke management trial with BP instead of stroke as the primary outcome due to the relatively short intervention duration of 1 year. Consistent results in systolic BP from sensitivity analyses and large effects on behavioral, functioning, and hard clinical outcomes were reassuring.

Quality of care improvement at the primary care level for chronic diseases management is a global challenge, especially for resource-limited settings. Rural China bears disproportionately high burdens of stroke, lacks capacity in delivering stroke care among community-dwelling patients, and encounters barriers in quality improvements at both the health system and individual levels [16,22]. Our study findings suggested that the benefit of the primary care mHealth-integrated intervention could be far beyond BP reduction alone, as demonstrated in the results on physical functioning, and even reduction in hospitalization and mortality. Although we were not able to pinpoint the mechanism of impact on these outcomes, the consistently significant results in stroke events, hospitalization, and mortality were potentially due to our practical and integrated intervention that addressed multiple domains. In line with previous literature [4,8,11], the training sessions, ongoing system-level technical support, and mHealth technological enablement improved the capacity of primary healthcare providers and the financial and nonfinancial incentives motivated them to provide evidence-based care. As the goalkeepers living within the community, providing monthly follow-up visits through village doctors was feasible and sustainable. The maintenance of the behavior changes was further strengthened through low-cost automated daily voice messages sent directly to patients. Such an intervention model is designed to be applicable to other NCDs and with high potential to be adapted to other LMICs settings for strengthening the capacity of the primary healthcare system in chronic disease management.

### Comparison with other studies

Different from other recent trials in LMICs that have shown the effectiveness of multicomponent interventions in hypertension and NCD control [4–6], our trial has 2 parallel features that synergistically supported each other and ensured the scalability and sustainability of the intervention. First, our primary care-based intervention was delivered by existing health workforce with system-level support and performance-based incentives. Instead of introducing new health workforces, we relied on primary care providers in the public sector who were already part of the primary care infrastructure. The quality of care they provided to stroke patients was improved through carefully designed evidence-based measures including training and ongoing support, mHealth technology, and incentives, which addressed existing challenges [23] and were in line with current guidelines [26].

Second, the intervention integrated provision of mHealth technology for both providers (village doctors and township physicians) and stroke patients. Compared to other stand-alone mHealth studies with inconsistent results [9,10], the SINEMA intervention was predominantly a primary care-based model emphasizing provider–patient interactions that were empowered via technologies. The Android-based App assisted village doctors by guiding follow-up visit procedures, collecting and storing related information, and managing the follow-up schedules and also shared the information with township physicians for quality control and monitoring. The step-to-step guidance and patients’ BP history and other information contained in the App may improve village doctors’ behaviors in prescribing evidence-based medicines and their communication with patients on adherence to treatment and lifestyle modifications. The automated voice messaging system dispatched a sizable amount of health education messages and reminders to patients that could not be achieved by traditional labor-intensive approaches. These voice messages provided suggestions and reminders on medication adherence and physical activities, which were consistent with the focus of the follow-up visits provided by village doctors and may reinforce the maintenance of behavior changes. The integrated mHealth system not only created new channels of communication and information flow in an effective and smart approach but also enhanced the effectiveness of human-delivered intervention components by removing barriers and reinforcing the maintenance of behavior changes among both providers and patients. Although we could not pinpoint the specific quantitative contributions of the integrated mHealth component in our study, our qualitative research provided evidence that the mHealth technology as an integral part of the package was well accepted and enhanced the effectiveness of the overall intervention.

Our intervention is different from previous studies as it targeted and reached a general village-dwelling population who had stroke for a relatively long term. Our participants had a median of 5 years duration living with stroke, 69.1% had been aware of having hypertension at baseline, and 37.2% self-reported that they were a current or former smoker. These characteristics of our participants are similar to those reported in the national registration of stroke survivors in rural China [41]. Such target population also distinguished our study from some of the existing hospital-oriented strategies of improving the care among stroke survivors who are recently discharged from the hospitals [41,42].

### Strengths and limitations

This trial has many strengths. First, it was designed on the bases of previous trials in rural China [12,13] and extensive field research for contextualization and adaption of the SINEMA model to local settings [15,18,19]. Second, it was rigorously implemented, including high recruitment rate with minimal loss to follow-up; satisfactory protocol fidelity; and independent standardized outcome assessment. Third, randomization and data analyses were performed by experienced biostatisticians in the United States who were not involved in trial implementation. Lastly, the report of study findings in this paper is in line with the prespecified and prepublished trial design [15] and statistical analysis plan [21].

Our study also has limitations. First, it was conducted in one province in Northern China, which may limit the generalizability of the study findings. However, our study population are similar to the national registered population, and our findings are relevant not only to rural China but other resource-limited settings as the intervention model is potentially feasible to scale-up to blood pressure or stroke management in other settings. Importantly, the adaptation of these strategies to local contexts is indispensable. For example, village doctors are best suited to deliver the intervention in rural China, while in other LMICs countries, collaborations between general practitioners and community health workers are necessary. We have been working with local and national governments in China to replicate the intervention with scale-up trial and also have assessed the feasibility of the intervention approach in other LMICs to provide more evidence on the generalizability [43–45]. Second, the trial was only 1 year long, and it was not powered for subgroup analyses, so larger studies are needed for better understanding of subgroup differences and long-term impacts. Third, we could not assess the independent contributions of each component and fully capture the mechanism of change due to the nature of the complex intervention design. We have conducted process evaluation through quarterly qualitative interviews among both providers and patients. Results from the process evaluation will be presented in a separate full-length manuscript to provide a deeper overview on the facilitators and barriers related to the intervention reach, adoption, implementation and effectiveness, and further explanation on the mechanism of impact. Although we have reported the cost of the delivery, we will also report cost-effectiveness modeling in another full-length manuscript.

### Implications for policy and future research

Our primary care-based integrated mHealth intervention, rigorously designed and implemented, resulted in a significant decrease in systolic BP, improvement in other health outcomes, and reduction in hospitalization and mortality among stroke patients in rural China. Chronic disease management requires continuous reliance on primary care, especially when the entire healthcare system is threatened by new global health crises such as the Coronavirus Disease 2019 (COVID-19). Our trial, conducted in the pre-COVID era, may have more important implications in the future as effort to strengthen primary care is needed to combat both NCDs and emerging infectious diseases. In LMICs where resources are limited or in low-resource areas in high-income countries, mHealth technology holds the potential to bring disruptive changes to health service delivery, quality improvement, and NCD control owing to its reach, convenience, cost efficiency, and lack of other traditional resources [8,9]. Thus, our SINEMA model, which is primary care-based, integrates provider-side and patient-side mHealth technology, and designed with the principles of sustainability and scalability, has the potential to be applied to other NCDs besides stroke and to other settings in China and globally. If demonstrated to be cost-effectiveness through full economic evaluation, the model may also have great potential to be adapted and scaled up to other settings to expand the health and economic benefits.

## Conclusions

Our primary care-based integrated mHealth intervention, rigorously designed and implemented, resulted in a significant decrease in systolic BP, improvement in other health outcomes, and reduction in hospitalization and mortality among stroke patients in rural China. Our low-cost intervention seamlessly combines service delivery by existing primary care workforce with mobile technology that integrates provider- and patient-side measures. If scaled up, it is expected to lead to large health and economic benefits in rural China and with adequate adaptations, potentially also in other resource-limited settings in both LMICs and high-income countries.

## Supporting information

S1 Checklist CONSORT checklist. (DOCX)

S1 Fig SINEMA intervention diagram. (TIF)

S1 Table Baseline characteristics for the SINEMA trial at the cluster and provider level. (DOCX)

S2 Table Baseline characteristics by status on loss to follow-up at the patient level. (DOCX)

S3 Table Fidelity to the intervention protocol in the 25 intervention villages. (DOCX)

S4 Table Program delivery costs over 12 months in US dollar. (DOCX)

S5 Table Sensitivity analysis of systolic blood pressure and diastolic blood pressure as hypertension control. (DOCX)

S6 Table Sensitivity analysis results on primary, secondary and exploratory outcomes. (DOCX)

## Abbreviations

BP: blood pressure
CI: confidence interval
LMICs: low- and middle-income countries
mHealth: mobile health
NCD: noncommunicable chronic disease
pp: percentage points
RD: risk difference
RR: risk ratio
SD: standard deviation
SINEMA: system-integrated and technology-enabled model of care
