# Supplementary figures and images for: Effectiveness of a primary care-based integrated mobile health intervention for stroke management in rural China (SINEMA): A cluster-randomized controlled trial
Source: PLoS Med. 2021 Apr 28;18(4):e1003582. doi: 10.1371/journal.pmed.1003582 (PMC8115798; doi:10.1371/journal.pmed.1003582)

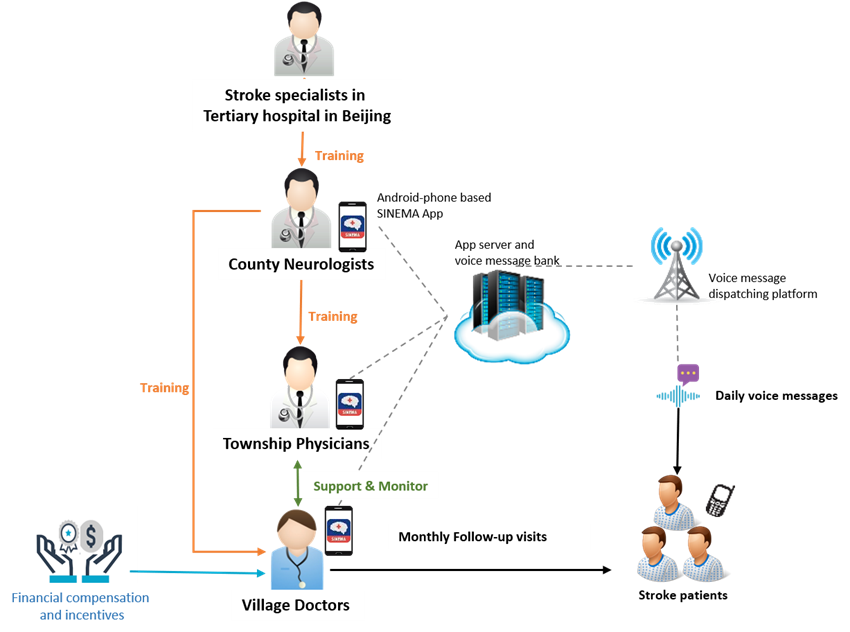

Supplement: S1 Fig — (TIF) [file pmed.1003582.s002.tif]
