# Supplementary material for: Effectiveness of a primary care-based integrated mobile health intervention for stroke management in rural China (SINEMA): A cluster-randomized controlled trial
Source: PLoS Med. 2021 Apr 28;18(4):e1003582. doi: 10.1371/journal.pmed.1003582 (PMC8115798; doi:10.1371/journal.pmed.1003582)
Supplement: S1 Table — (DOCX) [file pmed.1003582.s003.docx]

**S1 Table. Baseline characteristics for the SINEMA trial at the cluster and provider level**

|  | **Control**  **N=25** | **Intervention N=25** | **Total**  **N=50** |
| --- | --- | --- | --- |
| **Village characteristics*** |  |  |  |
| **Number of households in each village, median (Q1, Q3)** | 714  (614, 1,138) | 687  (594, 998） | 703.5  (594, 1,105) |
| **Number of residents in each village, median (Q1, Q3)** | 2458  (1,762, 3,650) | 2289  (1,787, 3,331) | 2422.5  (1,772, 3,600) |
| **Distance between village clinic to county hospital, km** | 14.3 (4.8) | 14.4 (4.9) | 14.5 (4.8) |
| **Number of stroke patients recruited, Min, MAX** | 21, 30 | 20, 30 | 20, 30 |
| **Number of recruited patients, n (%)** |  |  |  |
| 20-23 | 5 (20.0%) | 8 (32.0%) | 13 (26.0%) |
| 24-27 | 9 (36.0%) | 9 (36.0%) | 18 (36.0%) |
| 28-30 | 11 (44.0%) | 8 (32.0%) | 19 (38.0%) |
| **Village doctor characteristics (1 per village)** |  |  |  |
| **Age, mean (SD), years** | 45.9 (5.5) | 46.1 (7.3) | 46.0 (6.4) |
| **Sex, % female** | 5 (20.0%) | 3 (12.0%) | 8 (16.0%) |
| **Education, n (%)** |  |  |  |
| College | 1 (4.0%) | 2 (8.0%) | 3 (6.0%) |
| Junior college | 10 (40.0%) | 8 (32.0%) | 18 (36.0%) |
| High school or equivalent | 14 (56.0%) | 15 (60.0%) | 29 (58.0%) |
| **Years as village doctor, mean (SD), years** | 24.3 (6.1) | 24.2 (8.6) | 24.3 (7.4) |

*Q1: lower quartile, Q3: upper quartile, SD: standard deviation
