# Supplementary material for: Effectiveness of a primary care-based integrated mobile health intervention for stroke management in rural China (SINEMA): A cluster-randomized controlled trial
Source: PLoS Med. 2021 Apr 28;18(4):e1003582. doi: 10.1371/journal.pmed.1003582 (PMC8115798; doi:10.1371/journal.pmed.1003582)
Supplement: S2 Table — (DOCX) [file pmed.1003582.s004.docx]

**S2 Table. Baseline characteristics by status on loss to follow-up at patient level**

|  | **Not lost** | **Lost**  **(inc. death)** | **Total** |
| --- | --- | --- | --- |
| **Total participants recruited, n** | 1226 | 73 | 1299 |
| **Intervention allocation** |  |  |  |
| Control, n (%) | 615 (50.2%) | 47 (64.4%) | 662 (51.0%) |
| Intervention, n (%) | 611 (49.8%) | 26 (35.6%) | 637 (49.0%) |
| **Demographic characteristics and disease history** |  |  |  |
| **Age, mean (SD), years** | 65.8 (8.0) | 64.4 (10.5) | 65.7 (8.2) |
| **Sex, n (%) female** | 533 (43.5%) | 20 (27.4%) | 553 (42.6%) |
| **Education, n (%)** |  |  |  |
| No schooling | 515 (42.0%) | 23 (31.5%) | 538 (41.4%) |
| Some schooling or primary school only | 359 (29.3%) | 28 (38.4%) | 387 (29.8%) |
| Above primary school | 352 (28.7%) | 22 (30.1%) | 374 (28.8%) |
| **Marital Status, n (%)** |  |  |  |
| Married | 1,013 (82.6%) | 62 (84.9%) | 1075 (82.8%) |
| Widowed, divorced or not married | 213 (17.4%) | 11 (15.1%) | 224 (17.2%) |
| **Had none of the listed assets, n (%)**† | 75 (6.1%) | 3 (4.1%) | 78 (6.0%) |
| **Phone ownership, n (%)*** |  |  |  |
| No phone (may have a shared phone) | 299 (24.4%) | 24 (32.9%) | 323 (24.9%) |
| Basic phone | 836 (68.2%) | 39 (53.4%) | 875 (67.4%) |
| Smartphone | 91 (7.4%) | 10 (13.7%) | 101 (7.8%) |
| **Smoking Status, n (%)*** |  |  |  |
| Current smoker | 205 (16.7%) | 16 (21.9%) | 221 (17.0%) |
| Former smoker | 239 (19.5%) | 23 (31.5%) | 262 (20.2%) |
| Never smoker | 782 (63.8%) | 34 (46.6%) | 816 (62.8%) |
| **Enrolled in NCD insurance benefits package, n (%)**‡ | 159 (13.0%) | 9 (12.3%) | 168 (12.9%) |
| **Stroke type, n (%)** |  |  |  |
| Ischemic | 1056 (86.4%) | 63 (86.3%) | 1119 (86.4%) |
| Hemorrhage | 166 (13.6%) | 10 (13.7%) | 176 (13.6%) |
| **Self-report diseases, n (%)** |  |  |  |
| Hypertension | 854 (69.7%) | 43 (58.9%) | 897 (69.1%) |
| Dyslipidemia | 492 (40.1%) | 27 (37.0%) | 519 (40.0%) |
| Diabetes | 209 (17.0%) | 7 (9.6%) | 216 (16.6%) |
| Heart Diseases | 119 (9.7%) | 5 (6.8%) | 124 (9.5%) |
| **Outcomes at baseline** |  |  |  |
| **Systolic blood pressure, mean (SD), mmHg*** | 146.2 (22.2) | 140.0 (25.1) | 145.9 (22.4) |
| **Diastolic blood pressure, mean (SD), mmHg** | 79.0 (11.6) | 76.9 (13.6) | 78.88 (11.7) |
| **Health-related quality of life, mean (SD), utility score**§ | 0.8 (0.2) | 0.77 (0.26) | 0.80 (0.22) |
| **Timed Up-and-go, n (%) with time of completion ≥ 14 second** ¶ | 634 (52.4%) | 37 (51.4%) | 671 (52.4%) |
| **Physical activity, median (quartile 1, quartile 3), MET minutes/week** | 1173.0  (346.5, 2646.0) | 924.0  (297.0, 2772.0) | 1164.0  (346.5, 2670.0) |
| **Medication use, n (%)** |  |  |  |
| Antiplatelet | 802 (65.4%) | 50 (68.5%) | 852 (65.6%) |
| Statin | 325 (26.5%) | 15 (20.5%) | 340 (26.2%) |
| Anti-hypertensive medicines | 976 (79.6%) | 54 (74.0%) | 1030 (79.3%) |
| **Adherence to medications, n (%)** †† |  |  |  |
| Antiplatelet | 511 (63.7%) | 26 (52.0%) | 537 (63.0%) |
| Statin | 208 (64.0%) | 8 (53.3%) | 216 (63.5%) |
| Anti-hypertensive medicines | 511 (63.7%) | 26 (52.0%) | 537 (63.0%) |
| **Moderate to severe disability, n (%)** ‡‡ | 327 (26.7%) | 25 (34.2%) | 352 (27.1%) |
| **Stroke hospitalization in the past year, n (%)** | 241 (19.7%) | 15 (20.5%) | 256 (19.7%) |

SD: standard deviation; MET: metabolic equivalents

* Observed significant differences in analyses without taking cluster into account (p<0.03) on these variables.

†. TV, refrigerator, air conditioner, and computer were listed as home assets in the questionnaire.

‡. NCD insurance package is only available for people enrolled in health insurance system and with severe chronic diseases, through which people could get reimbursement of outpatient services at county hospital.

§. Health-related quality of life was measured by using EQ5D-5L and was converted into a utility score based on the Chinese value set.

¶. “Timed up and go” test results were recorded in seconds during measurement and dichotomized into binary as ≥14 (indicating lower mobility and more likely of fall) vs. <14s (higher mobility) based on previous literature.

††. Medication adherence was only measured among participants who were taking medicines based on 4-item Morisky Green Levine Scale.

‡‡. Disability was measured by modified Rankin Scale and people who received a score above three were grouped into the "moderate to severe disability" group.
