# Supplementary material for: Effectiveness of a primary care-based integrated mobile health intervention for stroke management in rural China (SINEMA): A cluster-randomized controlled trial
Source: PLoS Med. 2021 Apr 28;18(4):e1003582. doi: 10.1371/journal.pmed.1003582 (PMC8115798; doi:10.1371/journal.pmed.1003582)
Supplement: S3 Table — (DOCX) [file pmed.1003582.s005.docx]

**S3 Table. Fidelity to the intervention protocol in the 25 intervention villages**

|  | **Summary*** | **Range** |
| --- | --- | --- |
| **Village doctors (N=25)** |  |  |
| **Pre-intervention training** |  |  |
| Participation in the initial intensive training, n (%) | 25 (100.0%) | NA |
| Participation in the refresher training, n (%) | 21 (84.0%) | NA |
| **Monthly patient follow-up visits during the intervention**† |  |  |
| Follow-up visits delivered per village, mean (SD), range | 291.5 (39.5) | 209-352 |
| Follow-up visits delivered per patient, mean (SD), range‡ | 11.9 (0.4) | 10.9-12.9 |
| **Adoption of the SINEMA app** |  |  |
| Number (%) of village doctors using the app every month | 25 (100.0%) | NA |
| Number of detected incorrect entries per month, mean (SD), range§ | 0.9 (1.5) | 0-6 |
| **Interaction in the social media support group** |  |  |
| Number of messages sent, mean (SD), range | 37.4 (14.8) | 17-77 |
| Number of suggestive messages, mean (SD), range | 5.4 (2.2) | 0-10 |
| **Participants (N=611)** ‡ |  |  |
| **Pre-intervention briefing session** |  |  |
| Villages holding village-wide briefing sessions, n (%) | 25 (100.0%) | NA |
| Number of patients participating in the briefing sessions, mean (SD) | 530 (86.7%) | NA |
| **Monthly follow-up visits during the intervention**† |  |  |
| Number (%) of patients received ≥ 12 follow-up visits | 553 (90.5%) | NA |
| Number of follow-up visit received per person, mean (SD), range | 11.9 (0.74) | 3-14 |
| **Daily cell phone voice messages**¶ |  |  |
| Number (%) had a phone and agreed to receive daily voice messages | 491 (80.4%) | NA |
| Average proportion answering the voice message on a given day, mean (SD), range, % | 49.7% (3.0%) | 42.0%-58.0% |

SD: standard deviation

*Data are n (%) or mean (SD), unless otherwise indicated.

† Information related to follow-up visits was calculated based on the SINEMA *app* database records.

‡. For the calculation with patient-level information, we excluded participants who were lost to follow-up (n=73).

§ Incorrect entries were screened by program manager and township physicians based on the information that village doctors entered in the SINEMA *app* during the first three months of the intervention. Feedbacks for incorrect entries were provided to village doctors for correction or attention.

¶ Voice message was calculated based on the available records (from Nov. 1st, 2017 to July 27^th^, 2018) from the message dispatching system, which linked with the SINEMA app.
