# Supplementary material for: Effectiveness of a primary care-based integrated mobile health intervention for stroke management in rural China (SINEMA): A cluster-randomized controlled trial
Source: PLoS Med. 2021 Apr 28;18(4):e1003582. doi: 10.1371/journal.pmed.1003582 (PMC8115798; doi:10.1371/journal.pmed.1003582)
Supplement: S4 Table — (DOCX) [file pmed.1003582.s006.docx]

**S4 Table. Program delivery costs over 12 months in US dollar**

| **Items in program delivery costs** | **Total annual cost of the intervention arm**  **(in US dollar) *** | **Annual per-capita cost**  **in US dollar**  **(n=637) *** |
| --- | --- | --- |
| **Provider-side labour cost** |  |  |
| Local project manager | 687.7 | 1.1 |
| Compensation for county specialists who provide training and support | 257.9 | 0.4 |
| Compensation for township physicians | 1075.8 | 1.7 |
| Compensation and financial incentives for village doctors | 10092.3 | 15.8 |
| **Patient-facing component cost** |  |  |
| Voice messages | 1534.3 | 2.4 |
| Printing materials | 325.2 | 0.5 |
| **Digital health system maintenance** |  |  |
| Servers and system maintenance | 1486.2 | 2.3 |
| **Sum** | **15459.5** | **24.3** |

* World Bank annual average middle exchange rate for US dollar to Chinese Yuan in 2018 (6.98) was applied.
