# Supplementary material for: Effectiveness of a primary care-based integrated mobile health intervention for stroke management in rural China (SINEMA): A cluster-randomized controlled trial
Source: PLoS Med. 2021 Apr 28;18(4):e1003582. doi: 10.1371/journal.pmed.1003582 (PMC8115798; doi:10.1371/journal.pmed.1003582)
Supplement: S5 Table — (DOCX) [file pmed.1003582.s007.docx]

**S5 Table. Sensitivity analysis of systolic blood pressure and diastolic blood pressure as hypertension control**

|  | **Timepoint** | | **Minimally Adjusted Model*** | | **Fully adjusted model**** | |
| --- | --- | --- | --- | --- | --- | --- |
|  | **Baseline** | **12-month** | **Prevalence Ratio (95% CI)** | **P-value** | **Prevalence Ratio (95% CI)** | **P-value** |
| **Participants with systolic BP under-control**† | | | | | | |
| Control arm | 262 (42.6%) | 298 (48.5%) | Reference | NA | Reference | NA |
| Intervention arm | 251 (41.1%) | 338 (55.3%) | 1.15 (1.05, 1.26) | 0.002 | 1.20 (1.11, 1.30) | <0.001 |
| **Participants with BP under-control** ‡ | | | | | | |
| Control arm | 252 (41.0%) | 285 (46.3%) | Reference | NA | Reference | NA |
| Intervention arm | 241 (39.5%) | 334 (54.7%) | 1.19 (1.08, 1.30) | <0.001 | 1.23 (1.13, 1.34) | <0.001 |

CI: confidence interval

* Pre-specified main analysis (minimally adjusted model): Adjusted for baseline outcome, township, sex and age.

** Sensitivity analysis (fully adjusted model): Adjusted for baseline outcome, township, sex, age, variables noted to be differential by treatment arm at baseline (baseline diastolic blood pressure, having hypertension, having none of the assets asked about, taking anti-hypertensive medications) and loss to follow-up (baseline systolic blood pressure, annual household income, type of phone owned and smoking status).

† Systolic BP (blood pressure) under-control was defined as participants’ systolic blood pressure <140mmHg based on the Chinese hypertension guideline.

‡ BP (blood pressure) under-control was defined as participants’ systolic blood pressure <140mmHg and diastolic blood pressure <90 mmHg based on the Chinese hypertension guideline.
