# Supplementary material for: Effectiveness of a primary care-based integrated mobile health intervention for stroke management in rural China (SINEMA): A cluster-randomized controlled trial
Source: PLoS Med. 2021 Apr 28;18(4):e1003582. doi: 10.1371/journal.pmed.1003582 (PMC8115798; doi:10.1371/journal.pmed.1003582)
Supplement: S6 Table — (DOCX) [file pmed.1003582.s008.docx]

**S6 Table. Sensitivity analysis results on primary, secondary and exploratory outcomes**

| **Outcomes** | **Adjusted for imbalance related to**  **loss to follow-up** * | | **Adjusted for baseline imbalance**** | | **Analysis with no outliers removed***** | |
| --- | --- | --- | --- | --- | --- | --- |
|  | **Estimates** †  **(95% CI)** | **P-value** | **Estimates** †  **(95% CI)** | **P-value** | **Estimates** †  **(95% CI)** | **P-value** |
| **Primary outcome** |  |  |  |  |  |  |
| Change in systolic blood pressure, mmHg | -2.9 (-4.8, -0.9) | 0.004 | -3.2 (-5.1, -1.3) | 0.002 | -3.0 (-4.9, -1.0) | 0.004 |
| **Secondary Outcomes** |  |  |  |  |  |  |
| Change in diastolic blood pressure, mmHg | -2.3 (-3.3, -1.3) | <0.001 | -2.3 (-3.3, -1.3) | <0.001 | -2.2 (-3.2, -1.2) | <0.001 |
| Change in the utility score of health-related quality of life ‡ | 0.04 (0.01, 0.06) | 0.007 | 0.04 (0.01, 0.06) | 0.007 | 0.06 (0.02, 0.10) | 0.003 |
| Timed-up and-go (time of completion ≥ 14 s) § | 0.86 (0.77, 0.98) | 0.019 | 0.87 (0.77, 0.98) | 0.023 | NA | NA |
| Change in physical activity, MET min/week | 534.6 (278.8, 790.4) | <0.001 | 522.0 (264.7, 779.3) | <0.001 | 1041.6 (572.6, 1510.5) | <0.001 |
| Medication use |  |  |  |  |  |  |
| Antiplatelets | 1.15 (1.07, 1.23) | <0.001 | 1.15 (1.07, 1.23) | <0.001 | NA | NA |
| Statins | 1.04 (0.82, 1.33) | 0.718 | 1.06 (0.85, 1.32) | 0.623 | NA | NA |
| Anti-hypertensives | 1.06 (1.01, 1.11) | 0.018 | 1.06 (1.01, 1.12) | 0.019 | NA | NA |
| **Exploratory Outcomes** |  |  |  |  |  |  |
| Stroke Recurrence | 0.45 (0.31, 0.66) | <0.001 | 0.46 (0.32, 0.67) | <0.001 | NA | NA |
| Stroke hospitalization in the past year | 0.66 (0.54, 0.80) | <0.001 | 0.66 (0.54, 0.81) | <0.001 | NA | NA |
| Moderate to severe disability ¶ | 0.45 (0.31, 0.64) | <0.001 | 0.45 (0.31, 0.65) | <0.001 | NA | NA |
| Death †† | NA | NA | 0.48 (0.25, 0.90) | 0.022 | NA | NA |

CI: confidence interval; MET: metabolic equivalents

* Adjusted for baseline outcome, township, gender and age, variables noted to be differential by treatment arm at baseline (based on p < 0.05; baseline diastolic blood pressure, having hypertension, having none of the assets asked about, taking anti-hypertensive medications); removing outliers in the outcome variable (based on a priori decision to remove those that are more than two interquartile range above the third quartile or below the first quartile).

** Adjusted for baseline outcome, township, gender, age, variables noted to be differential by loss to follow-up (based on p < 0.05; baseline systolic blood pressure, annual household income, type of phone owned and smoking status); removing outliers in the outcome variable (based on a priori decision to remove those that are more than two interquartile ranges above the third quartile or below the first quartile).

*** Fully adjusted model but without the outliers removed. Adjusted for baseline outcome, township, sex, age, variables noted to be differential by treatment arm at baseline (baseline diastolic blood pressure, having hypertension, having none of the assets asked about, taking anti-hypertensive medications) and loss to follow-up (baseline systolic blood pressure, annual household income, type of phone owned and smoking status).

† For continuous outcomes (systolic blood pressure, diastolic blood pressure, EQ5D-5L, physical activity), “estimate” refers to the differences between the arms in mean one-year change in the outcome (control arm is the reference); for categorical outcomes, “estimate” refers to the prevalence ratio (control arm is the reference).

‡. Health-related quality of life was measured by using EQ5D-5L and was converted into a utility score based on the Chinese value set.

§. “Timed up and-go test” results were recorded in seconds during measurement and dichotomized into binary as ≥14 (indicating lower mobility, higher possibility of fall) vs. <14s based on previous literature.

¶. Disability was measured by modified Rankin Scale and people who received a score above 3 were grouped into the "moderate to severe disability" group.

††. The statistical model with death as the outcome was not adjusted for variables differential by loss to follow-up, since those who died during the study are a subset of the group lost to follow-up.
